# Evaluation of the pathogenicity of West Nile virus (WNV) lineage 2 strains in a SPF chicken model of infection: NS3-249Pro mutation is neither sufficient nor necessary for conferring virulence

**DOI:** 10.1186/s13567-015-0257-1

**Published:** 2015-10-30

**Authors:** Maha Dridi, Thierry Van Den Berg, Sylvie Lecollinet, Benedicte Lambrecht

**Affiliations:** Operational Direction of Viral Diseases, CODA-CERVA-Veterinary and Agrochemical Research Centre, 99 Groeselenberg, 1180 Brussels, Belgium; UPE, UMR1161 Virologie, Institut National de la Recherche Agronomique (INRA), Agence Nationale de Sécurité Sanitaire de l’alimentation, de l’environnement et du travail (ANSES), Ecole Nationale Vétérinaire d’Alfort (ENVA), 14 rue Pierre et Marie Curie, 94701 Maisons-Alfort, France

## Abstract

Lineage 2 West Nile virus (WNV) strains were reported for the first time in Europe in 2004. Despite an almost silent circulation around their entry point in Hungary, an upsurge of pathogenicity occurred in 2010 as 262 people suffered from neuroinvasive disease in Greece. This increase in virulence was imputed to the emergence of a His249Pro mutation in the viral NS3 helicase, as previously evidenced in American crows experimentally infected with the prototype lineage 1 North-American WNV strain. However, since 2003, WNV strains bearing the NS3Pro genotype are regularly isolated in Western-Mediterranean countries without being correlated to any virulent outbreak in vertebrates. We thus sought to evaluate the weight of the NS3_249Pro_ genotype as a virulence marker of WNV in an in vivo avian model of WNV infection. We therefore characterized three genetically-related Eastern-Europe lineage 2 WNV strains in day-old specific pathogen-free (SPF) chickens: Hun2004 and Aus2008 which are both characterized by a NS3_249His_ genotype, and Gr2011 which is characterized by a NS3_249Pro_ genotype. Unlike Hun2004 and Aus2008, Gr2011 was weakly virulent in SPF chicks as Gr2011-induced viremia was lower and waned quicklier than in the Hun2004 and Aus2008 groups. Overall, this study showed that the presence of a proline residue at position 249 of the viral NS3 helicase is neither sufficient nor necessary to confer pathogenicity to any given lineage 2 WNV strain in birds.

## Introduction

West Nile virus (WNV) is a mosquito-borne, positive sense, single-stranded RNA virus that belongs to the *Flavivirus* genus (*Flaviviridae* family), and that falls within the Japanese encephalitis virus antigenic complex [1]. WNV is maintained in a bird-arthropod cycle, with mosquitoes of the *Culex* genus representing its main vector, and wild birds the principal hosts [2, 3]. Nevertheless, incidental infections of reptiles, amphibians and mammals are also described, with humans and equines being the most susceptible to the disease [1, 4].

Phylogenetic studies of pathogenic WNV strains segregate the isolates in two main lineages: lineage 1, which is composed of WNV strains from Europe, the Middle East, North America, Africa, Eastern Asia and Australia, and lineage 2, which contains strains historically isolated in sub-Saharan Africa and Madagascar and that were initially considered non-pathogenic in humans and horses [5, 6]. Nevertheless, in 2004, a WNV strain related to Central African lineage 2 viruses was isolated from a sick goshawk (*Accipiter gentilis*) in Hungary, and WNV-induced encephalitis was diagnosed in two extra goshawks and two sparrow hawks (*Accipiter nisus*) in 2005 [7]. Until 2007, sporadic cases were diagnosed in Hungary in wild birds, sheep, horses and humans, but between 2008 and 2009, lineage 2 WNV quickly spread westward to neighboring Austria, and was responsible in both countries for an increased mortality for raptors and disease involving the central nervous system (CNS) in 18 horses and 22 humans [8–10]. A lineage 2 Hungarian isolate from 2010 was further experimentally characterized in intraperitoneally-inoculated C57BL/6 mice where it exhibited low LD50 values (10^−0.48^ TCID_50_) and high RNA loads in the brain [11]. The Austro-Hungarian lineage 2 virus subsequently spread southward to the Thessaloniki Greek region where an impressive WN neuroinvasive disease (WNND) epidemic broke out in humans in 2010, affecting 262 people amongst which 34 succumbed [12, 13]. The same year, the first human outbreak of neuroinvasive disease due to a lineage 2 WNV was reported in Romania [14]. This outbreak was caused by a strain that was highly similar to a Volgograd lineage 2 strain involved in an outbreak in Russia in the Volga Delta area in 2007, but is unrelated to the Greek-Hungarian lineage 2. For this reason, lineage 2 WNV strains are supposed to have been introduced in Europe from Africa in at least two independent occasions [15]. In 2011, a lineage 2 WNV strain related to the Hungarian-Greek strains was reported for the first time in a dead Eurasian collared dove (*Streptopelia decaocto*) and in *Culex pipiens* mosquitoes in North-Eastern Italy and also in at least two febrile patients from Central Italy and Sardinia [16–18] and caused at least 7 human cases of WNND in 2013 in the Po river area [19–21]. WNV lineage 2 strains related to the Greek-Hungarian cluster have more recently spread to Serbia, where a large human outbreak occurred in 2012 [22, 23]. Finally, four lineage 2 isolates closely related to the Austrian, Italian and Serbian strains reported in 2008, 2011 and 2012 respectively, were detected in mosquitoes in the Czech Republic during 2013 [24].

The Eastern-European lineage 2 WNV strain responsible for the major human outbreak that took place in Greece in 2010 was characterized by the presence of a NS3_249Pro_ genotype [13, 19]. Similarly, the most severe European and Mediterranean WNV epidemics caused by lineage 1 WNV strains (Romania in 1996, Israel in 1998 and Volgograd in 1999) were also due to strains bearing the NS3_249Pro_ genotype [25, 26]. Moreover, the vast majority of WNV strains isolated in North America—where WNND took its heaviest toll worldwide—are characterized by a NS3_249Pro_ genotype [27]. This association between enhanced pathogenicity for birds and humans and the NS3_249Pro_ genotype led to proposing this mutation as a molecular determinant of WNV pathogenicity. Experimentally, birds are the only hosts so far where the NS3_249Pro_ substitution was demonstrated to be a virulence marker of WNV by means of mutational analyses using the genetic backbone of the prototype North-American lineage 1 WNV strain NY99 [25]. Indeed, American crows (*Corvus brachyrhynchos*) and House sparrows (*Passer domesticus*) exhibited higher viral loads and/or mortality rates when position 249 of the NS3 was occupied by a proline residue rather than by a threonine, alanine or asparagine, while 3 week-old CD-1 mice showed comparable LD50 values and neurovirulence phenotypes no matter the inoculated variant [25, 26]. Importantly, since 2003, lineage 1 WNV strains bearing the NS3_249Pro_ genotype are regularly isolated from Western-Mediterranean countries, like Italy, Spain and Portugal, without being correlated to any significant virulent outbreaks in humans, horses, nor birds, or inducing an enhanced pathogenicity in experimentally infected birds and mice [28–30].

On the one hand, if WNV E protein glycosylation on residue 154 clearly appears as being an undeniable and versatile virulence determinant of WNV that enhances infection in birds as well as in mice and *Culex* mosquito species [31–36], the presence of a proline residue at position 249 of the viral NS3 helicase is so far ambiguous as of its role in increasing WNV virulence in any given WNV strain. On the other hand, SPF chickens aged 1 day at the time of an experimental inoculation with WNV were previously shown to represent a sensitive avian model for WNV infection as well as a reliable tool for pathotyping epidemiologically different WNV strains [37]. Therefore, we sought to compare the pathogenicity of genetically close lineage 2 WNV strains (the Hungarian Hun2004, the Austrian Aus2008 and the Greek Gr2011) that bear differing residues at position NS3_249_ (proline or histidine) [19] in a SPF chicken model for WNV infection. The aims of this study were to investigate whether (1) three lineage 2 West Nile virus strains associated to different epidemiologies in the field would elicit different pathotypes in the day-old SPF chicken infection model, and whether (2) this in vivo characterization would infirm or confirm the NS3_249Pro_ genotype as a virulence marker of lineage 2 West Nile virus strains.

## Materials and methods

### Viruses and virus preparations

Lineage 2 WNV strains Hun2004 (genotype NS3_249His_, Genbank accession no: DQ116961.1), Aus2008 (genotype NS3_249His_, Genbank accession no: KF179640.1) and Gr2011 (genotype NS3_249Pro_, Genbank accession no: JN398476.2) were used. The inocula were prepared by amplification in Vero cells (African green monkey kidney-derived cells, provided by P. Desprès from the Pasteur Institute of Paris), for 2 passages for Hun2004 and Aus2008, and for 3 passages for Gr2011. Infected cells were analyzed by a freeze–thaw cycle after each passage and both virus-containing cell lysates and supernatants were frozen and stored in aliquots at −80 °C.

One aliquot from each amplified virus was used to titrate the strains using the TCID_50_ titration method [37]. TCID_50_/mL values were calculated according to the Reed and Muench method [38].

### Chickens

After hatching, SPF white Leghorn chickens provided by Lohmann Valo (Cuxhaven, Germany) were kept in biosecurity level 3 (BSL-3) isolators and animal experiments were conducted under the authorization and supervision of the Biosafety and Bioethics Committees at the Veterinary and Agrochemical Research Institute, following National and European regulations (procedure agreement no 111202-01). The birds were provided with a commercial diet for poultry and water ad libitum throughout the experiments.

### Experimental inoculations

In independent experiments, SPF chickens were inoculated subcutaneously (100 µL inoculum/chick) in the cervical region at the age of 1 day with a dose of 10^3^ TCID_50_ of West Nile virus diluted in sterile PBS. The dose of 10^3^ TCID_50_ was chosen because it is close to the dose usually inoculated by *C. pipiens* mosquitoes during natural infections (i.e. 10^4.3^ PFU, [39]), and because we showed in a previous work [37] that this dose allows a significant discrimination between different pathotypes of lineage 1 West Nile virus strains in the SPF chicken model, based on mortality rates. The number of inoculated chicks amounted to 52 for Hun2004, 51 for Aus2008 and 42 for Gr2011. On 2, 5, 7, 9, 12 and 14 days post-infection (dpi), 5 chickens per sub-group were sacrificed by exsanguination. Blood was immediately collected in microtubes and allowed to clot for 6 h at room temperature (RT°). After centrifugation at 2345* g* for 5 min, serum was collected and aliquots stored at −20 or −80 °C for further determination of neutralizing antibodies titers or RNA extraction, respectively. Two to three wing primary feathers were sampled from every sacrificed bird and stored in 600 µL of RNA later (RNA Stabilization Reagent, Qiagen Benelux B.V., The Netherlands) at −80 °C for further RNA extraction.

### Viremia monitoring

RNA was extracted from sera samples using a QIAamp Viral RNA Mini Kit (Qiagen Benelux B.V., Venlo, The Netherlands) according to the manufacturer’s instructions. RNA was recovered in 60 µL elution buffer (provided by the manufacturer) and stored at −80 °C. Two µL of each extract, in 25 µL final volume, were subjected to rRT-PCR amplification using the QuantiTect Probe RT-PCR Kit (Qiagen GmBh, Hilden, Germany) and following manufacturer’s instructions. The mix included primers (WNNS2-F: 5′-CCTTTTCAGTTGGGCCTTCTG-3′ and WNNS2a-R: 5′-GATCTTGGCTGTCCACCTCTTG-3′) at 0.15 µM final concentration, and a FAM-labeled TAMRA probe (WNNS2A-6-FAM TAMRA: 5′-TTCTTGGCCACCCAGGAGGTC-3′-TAMRA) at 0.2 µM final concentration. Amplification conditions consisted of a first reverse-transcription step at 50 °C for 30 min, followed by 10 min at 95 °C and 50 cycles of 15 s at 95 °C, 34 s at 54 °C and 10 s at 72 °C, as previously described [37]. Quantitation of WNV RNA copy number was calculated by generating standard curves with serial dilutions of quantified WNV NS2a synthetic RNA.

### Viral genome detection in feathers

Feathers were prepared by homogenization for 6 min at 30 cycles/s in 600 µL of Ambion^®^ MagMAX™ Lysis⁄Binding Solution Concentrate (Life Technologies Europe B.V., Gent, Belgium) 0.04 M DTT using a TissueLyzer™ homogenizer (Qiagen, Venlo, The Netherlands) and in presence of a 5 mm Ø stainless steel bead. After clarification at 8000 rpm for 10 min, RNA was extracted using a MagMAX™-96 Total RNA Isolation Kit (Life Technologies Europe B.V., Gent, Belgium) according to the manufacturer’s instructions. RNA was recovered in 50 µL of elution buffer (provided by the manufacturer) and stored at −80 °C. Two µL of each extract were subjected to the NS2a-specific rRT-PCR in 25 µL final volume.

### Neutralizing antibody titers determination

β-seroneutralization (SN) allows the titration of the neutralizing antibodies present in a serum by diluting the latter while keeping a constant concentration of virus. The diluted serum and the constant dose of virus are brought into contact with competent cells that are then plated in a multiwell plate. The neutralizing antibodies titer is deduced from the last serum dilution where the competent cells do not show cytopathic effects (CPE). Every serum sample was inactivated during 30 min at 56 °C and tested in duplicate. Sera were subjected to a series of threefold dilutions beginning from a 1/5 dilution in DMEM High Glucose Pyruvate solution (Life Technologies Europe B.V., Belgium) supplemented with 5.6 mg/L of Tylosin Solution (Sigma-Aldrich, Steinheim, Germany) in a final volume of 50 µL, and were subsequently transferred to a flat bottom 96-well plate (Nuncleon Delta Surface, Thermo Fischer Scientific, Roskilde, Denmark). 10^2^ TCID_50_/mL of the WNV strain used for experimental inoculation, in a volume of 50 µL, were then added in every serum-containing well. After 1 h 30 min of incubation at 37 °C under 5% CO_2_, 100 µL of a 1/20 dilution of freshly passaged Vero cells were added to every well. After three further days of incubation at 37 °C under 5% CO_2_, wells were visually assessed for the development of cytopathic effects, and the mean titer in neutralizing antibodies was calculated for every serum sample.

### Statistical analysis

Statistical analyses of data were performed using Minitab 13 and STATA 10 software (statistical programs for Windows 2000) and differences were considered significant at *P* < 0.05.

For each dpi, viremia values after inoculation with Hun2004, Aus2008 and Gr2011 were compared. After checking the validity hypotheses (normality of the criterion distribution for each group by the Ryan–Joiner test and homogeneity of the within-groups’ variances by the Levene test), ANOVA and Tukey’s pairwise comparison tests were used for comparison of the three Hun2004, Aus2008 and Gr2011 groups. When normality or homogeneity of variance tests failed, the non-parametric Mann–Whitney test on the one hand, and Kruskal–Wallis test on the other hand, were used. Similar statistical analyses were performed on the detection of neutralizing antibodies in serum and NS2a RNA in feathers in order to compare the three sets of inoculated groups at different dpi.

## Results

### Virological characterization

**Figure 1:**
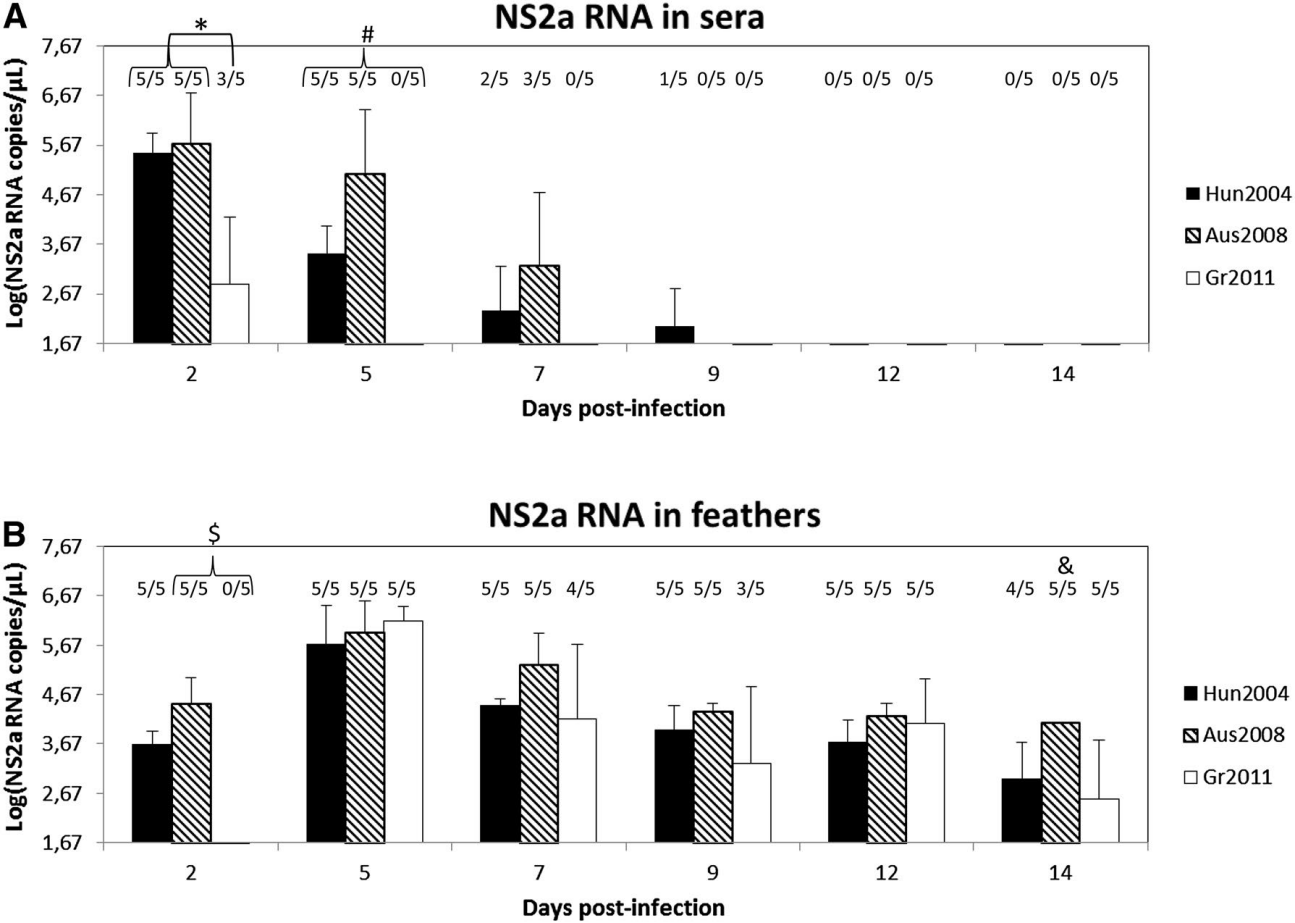
**Viral RNA loads follow-up in sera and feathers after infection with lineage 2 WNV strains.** Mean viral loads in sera **A** and feathers **B** were estimated by NS2a-specific rRT-PCR in day-old SPF chicks experimentally inoculated with either Hun2004 (black bars), Aus2008 (hatched bars) or Gr2011 (white bars). Error bars represent the standard error of the mean. Above the bars, numbers of positives/examined samples are indicated. *P < 0.05 for Hun2004 and Aus2008 versus Gr2011 at 2 dpi. ^#^ *P* < 0.05 for Hun2004 versus Aus2008 versus Gr2011 at 5 dpi. ^$^ *P* < 0.05 for Aus2008 versus Gr2011 at 2 dpi. ^&^ *P* < 0.05 for Aus2008 versus Hun2004 and Gr2011 at 14 dpi. NS2a-specific rRT-PCR detection limit is 10^1.67^ RNA copies/µL.

SPF chickens were inoculated subcutaneously at the age of 1 day with a dose of 10^3^ TCID_50_ of Hun2004, Aus2008 or Gr2011 and experimental follow-up was carried out for 14 dpi. Viremia—as measured by the log of NS2a RNA copies/µL—was detectable from 2 to 9 dpi and from to 2 to 7 dpi after inoculation of chickens with Hun2004 and Aus2008 strains respectively (Figure 1A). Viremia peaked at 2 dpi whatever the viral strain, and decreased steadily all over the detection period. However, Gr2011 induced a detectable viremia only on 2 dpi. Viremia evolution profiles did not only differ in duration but also in amplitude, as viremia at 2 dpi was significantly higher (*p* < 0.05) in the Hun2004 and Aus2008 groups than in the Gr2011 group. Moreover, at 5 dpi, the Aus2008 group had the highest viremia (*p* < 0.05) while the Gr2011 had the lowest viremia (*p* < 0.05). Overall, the viral load was statistically significantly higher (*p* < 0.05) in the Aus2008 and Hun2004 groups than in the Gr2011 group.

In feathers, viral genome was detectable all over the infection period, from 2 to 14 dpi for both Hun2004 and Aus2008 viral strains (Figure 1B). In chickens inoculated with Gr2011, viral load in feathers was also detectable up to 14 dpi, but the rRT-PCR gave positive results beginning from 5 dpi only. With the three viral strains, RNA detection in feathers reached a peak at 5 dpi, and only slightly decreased over the infection. Overall, Aus2008 elicited significantly higher (*p* < 0.05) viral loads in feathers than Gr2011 all over the infection period. Moreover, when data were examined dpi by dpi, Aus2008 elicited significantly higher (*p* < 0.05) viral loads in feathers than Gr2011 at 2 dpi, and than Hun2004 and Gr2011 at 14 dpi.

### Serological characterization

**Figure 2:**
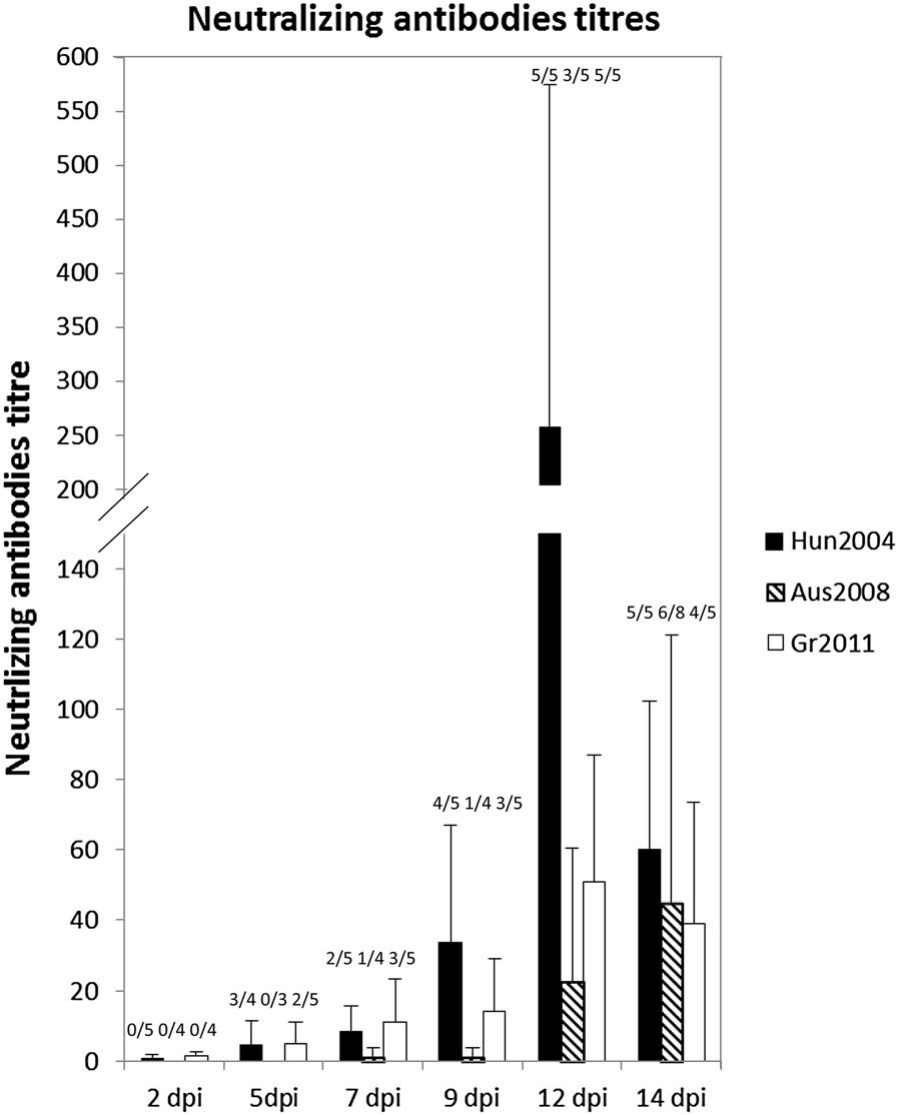
**Neutralizing antibodies titers follow-up after infection with lineage 2 WNV strains**. Mean neutralizing antibodies titers over days post-infection were estimated by β-seroneutralization tests in day-old SPF chicks experimentally inoculated with either Hun2004 (black bars), Aus2008 (hatched bars) or Gr2011 (white bars) lineage 2 WNV strains. Error bars represent the standard error of the mean. Above the bars, numbers of positives/examined samples are indicated.

The three lineage 2 WNV strains induced seroconversion in the inoculated chicks (Figure 2). Neutralizing antibodies were first detected in chickens inoculated with Hun2004 and Gr2011, at 5 dpi, and then in chicks infected with Aus2008, at 7 dpi. The overall humoral response was amplest in chicks of the Hun2004 group, but this difference was not statistically significant.

## Discussion

We have compared in this study the pathogenic profile of related lineage 2 WNV strains bearing either a NS3_249Pro_ or a NS3_249His_ genotype, while previous studies dealing with NS3_249_ polymorphism usually compared NS3_249Pro_ genotype with NS3_249Thr_ genotype [25, 28, 30, 40]. We have shown that despite its NS3_249Pro_ genotype, Gr2011 was weakly pathogenic in a SPF chicken model of WNV infection. Indeed, Gr2011-induced viremia was lower and waned quicklier than in the Hun2004 and Aus2008 groups. The study by Langevin et al. [26] has shown that even if the scientific literature tends to oppose the NS3_249Pro_ genotype against all the other existing NS3_249_ polymorphisms, the alternative residues alanine, threonine, histidine and asparagine are not equally less efficient than proline in enhancing virulence in experimental settings [26]. Thus, at least in vitro and in American crows, a histidine residue at position 249 of the NS3 of the North-American lineage 1 prototype strain NY99 is less efficient than a threonine residue at decreasing its initial virulence, which is due to its NS3_249Pro_ genotype. This has also been shown through our experimental inoculations of SPF chickens with three lineage 2 WNV strains which either bear a NS3_249His_ genotype (Hun2004 and Aus2008) or a NS3_249Pro_ genotype (Gr2011). These strains belong to the Greek-Hungarian cluster of lineage 2 WNVs [8]. Moreover, Hun2004 and Aus2008 strains are 99% identical with regards to their nucleotide as well as to their amino-acid sequences [8]. Aus2008 and Hun2004 are both characterized by a NS3_249His_ genotype, and they only diverge as of four residues located in the E, NS1, NS2a and NS5 viral proteins [19] (Table 1). In accordance with their NS3_249His_ genotype, Aus2008 and Hun2004 were both virulent in the SPF chicken model. Aus2008 was experimentally inoculated by Del Amo et al., to House sparrows (*P. domesticus*) which comparably developed detectable viremia from 1 to 5 dpi with a peak at 3 dpi [40]. Moreover, an experimental infection study using an Austrian lineage 2 WNV isolate from 2009 showed that one-third of the assayed large falcons (*Falco rusticolus*, and *F. rusticolus* × *F. cherrug* and *F. rusticolus* × *F. peregrinus* hybrids) succumbed to the virus while all the birds developed viremia and shed virus [41]. The pathogenicity of Hun2004 and Aus2008 for chickens evidenced the fact that the presence of a proline residue at position 249 of the NS3 helicase is not necessary for a given WNV isolate to be virulent in infection models. Indeed, unrelated lineage 1 strains that bear a threonine instead of a proline at position 249 of the NS3 have also been associated with deadly epidemics in humans and horses in Tunisia, Italy, France and Spain in respectively 1997, 1998 and 2008, 2000 and 2010 [5, 25, 42–44].

**Table 1:** Variant amino acids encoded by WNV lineage 2 genome sequences of Hun2004, Aus2008 and Gr2011

Genome sequence name (Genbank accession number)	Mature peptide								
	Amino acid position in mature peptide (Amino acid position in polyprotein)								
	E	NS1	NS2a	NS2b	NS3	NS4b			NS5
	88 (378)	69 (860)	1 (1140)	119 (1493)	249 (1754)	14 (2287)	49 (2322)	113 (2386)	25 (2554)
Hun2004 (DQ116961)	Ser	Gly	His	Val	His	Ser	Thr	Val	Ala
Aus2008 (KF179640)	Pro	Glu	Tyr	Val	His	Ser	Thr	Val	Thr
Gr2011 (JN398476)	Pro	Glu	Tyr	*Ile*	*Pro*	*Gly*	*Ala*	*Met*	Thr

Residues that are unique to Gr2011 when compared to Hun2004 and Aus2008 are italicized. (Adapted from [19, 46] and from a personal communication from Sylvie Lecollinet and Céline Bahuon (Anses))

The Gr2011 strain is directly derived from and genetically almost identical to the Nea Santa-Greece-2010 strain responsible for the dramatic 2010 Greek human epidemic [45–47]. Even though Hun2004 and Aus2008 are genetically highly related to their Greek progeny strains Gr2011 and Nea Santa-Greece-2010, the Greek strains cluster in a distinct phylogenetic group and are characterized by a NS3_His249Pro_ substitution [46]. Although the experimental infection of European Jackdaws (*Corvus monedula*) showed a high sensitivity of the assayed birds to a lineage 2 strain circulating in 2010 in Greece [48], notable avian mortalities associated with the Greek lineage 2 isolates were absent in the field. Accordingly, Gr2011 was in this study the least virulent strain in the SPF chicken infection model. Gr2011 indeed elicited only a low and short-term viremia (detection limited to 2 dpi). This observation, in addition to the association of lineage 2 Greek strains with human WNND cases, highlights the fact that avian WNV infection models might be predictive of the pathotype of a given WNV in birds only, and not in mammalian hosts. Nevertheless, given the central role of birds in amplifying and transmitting WNV to potential ornithophilic or bridge vectors, their susceptibility to be infected by a given WNV strain and to develop high-titer viremia following infection is positively correlated to their ability to spread the virus in an enzootic and zoonotic manner [49]. Of note, despite general low virulence, Gr2011 induced detectable viremia in SPF chicks at 2 dpi, underlining its potential amplification and spread in avian hosts.

In our hands, WNV RNA was detected in the feathers of all three experimental groups infected with Hun2004, Aus2008 or Gr2011 up to the end of the observation period (i.e., 14 dpi). This slower clearance of WNV from the feathers compared to serum was previously observed in the SPF chickens [37]. Nevertheless, the levels of detected viral RNA loads in the feathers still correlated with viremia levels. Indeed, viral RNA loads in the feathers of the Aus2008 group were significantly highest over the whole infection period, while viremia of the same group showed the highest peak at 5 dpi. Moreover, viral RNA load detection in the feathers of the GR2011 group was delayed to 5 dpi, while viremia in this group waned the fastest, as virus was detected in the serum of the GR2011 group only on 2 dpi.

The low virulence of Gr2011 for day-old SPF chicks in our hands highlighted the fact that the presence of a proline at position 249 of the NS3 helicase does not guarantee an enhanced virulence in a given infection model. Accordingly, an Italian strain isolated in 2012, also bearing a proline in position 249 of NS3 and found to be exactly similar to Nea Santa-Greece-2010 after the alignment of their NS5 and NS3 partial sequences, was associated with neither human nor veterinary cases in the region where the strain had been isolated [21]. By inoculating mice, red partridges (*Alectoris rufa*) and house sparrows (*P. domesticus*) with related strains bearing or not a NS3_249Pro_ genotype, Del Amo et al. and Sotelo et al. also came to the conclusion that a proline residue at position 249 of NS3 is neither sufficient nor necessary for WNV pathogenicity [28–30, 40]. Additionally, attention must be brought to the fact that Gr2011 not only differs from both Aus2008 and Hun2004 as of the residue at position 249 of NS3, but also at the level of residues located at positions 119 of NS2b and 14, 49 and 113 of NS4b (Table 1). Within Gr2011 genetic backbone, complementarily to or independently from the NS3_His249Pro_ substitution, one or more of these four additional substitutions might then be responsible for the loss of pathogenicity of Gr2011 for raptors [8] and SPF chickens, and/or for its increased virulence for humans compared to Hun2004 and Aus2008. Mutational analyses of NS2b, the co-factor of the NS3 protease and a protein implicated in the formation of viral replication complexes [50–52], are not described in the literature. However, such studies are available for NS4b. WNV NS4b co-localizes with other components of the viral replication complex, such as NS3 and double-stranded RNA [52] and is critical for the induction of paracrystalline arrays/convoluted membranes and vesicle packets allowing for replication, membrane complex formation and maturation of viral proteins [52–54]. Moreover, NS4b inhibits the interferon-signaling cascade at the level of nuclear STAT phosphorylation [55]. Remarkably, NS4b has a highly conserved N-terminal motif (residues 35 through 60) among Flaviviruses and a central hydrophobic region (residues 95 through 120). Substitutions brought simultaneously in the N-terminal domain and the central hydrophobic region of NS4b (Pro38Gly and Thr116Ile), or occurring in the central hydrophobic region alone (Cys102Ser) of a clone of NY99, correlated with an attenuated neuroinvasive phenotype in mice and a small plaque variant [56, 57]. One could then speculate that at least one or both of the two NS4b_Thr49Ala_ and NS4b_Val113Met_ substitutions, which occurred within Gr2011 strain in respectively the N-terminal and central hydrophobic region, could have modified the pathogenicity of Gr2011 when compared to Hun2004 and Aus2008, despite the presence of the NS3_249Pro_ genotype. However, this hypothesis demands extensive experimental studies in various species to be evaluated.

We chose in this study to use SPF chickens as an infection model as in the particular context of West Nile disease, they belong to a taxon (*Aves*) that represents the natural reservoir of the virus. Moreover, SPF chickens as experimental animals present many advantages over other vertebrates as they are easy to breed, genetically homogeneous, and free from maternally-derived antibodies and from specific pathogens and other underlying diseases [58–60]. Finally, we have shown in a previous study that SPF chickens aged 1 day at the time of experimental inoculation not only represent a sensitive avian model for WNV infection, but also allow the pathotyping of epidemiologically varying WNV strains [37].

In this study, we sought to demonstrate the extent to which the presence of a proline residue at position 249 of the viral NS3 helicase constitutes a determinant of virulence for given European lineage 2 isolates with amino-acid sequences differing in more than one location. On the one hand, it appeared that the presence of a proline residue at position 249 of the NS3 of the studied strains is not a necessary or sufficient determinant of virulence for SPF chickens. On the other hand, it seemed that the NS3_249His_ substitution might not have such an attenuating effect on WNV virulence as the NS3_249Thr_ residue. We indeed showed in the SPF chicken model that the two tested lineage 2 WNV strains bearing a Histidine at position 249 of the NS3, namely Hun2004 and Aus2008, elicited higher and longer-lasting viremias than the Gr2011 strain bearing a Proline at position 249 of NS3. Moreover, our finding that the Gr2011 strain was the least virulent in the SPF chickens model despite its high virulence for humans highlighted the predictability of this avian model for pathogenicity in birds specifically, as Greek lineage 2 WNV strains did not associate so far with notable bird mortalities in the field. This makes the SPF chicken model a promising surrogate for experimental inoculations in wild birds.

## Abbreviations

CNS: central nervous system
CPE: cytopathic effect
RT: room temperature
SN: seroneutralization
SPF: specific pathogen free
TCID_50_: tissue culture infectious dose 50
WNND: West Nile neuroinvasive disease
WNV: West Nile virus
